# Facilitating pain assessment and communication in people with deafness: a systematic review

**DOI:** 10.1186/s12889-023-16535-5

**Published:** 2023-08-22

**Authors:** Irene Mansutti, Catarina Tomé-Pires, Stefania Chiappinotto, Alvisa Palese

**Affiliations:** 1 Bachelor of Nursing, Department of Medical Science, Udine University, Viale Ungheria 20, 33100 Udine, Italy; 2 Psychology Research Centre (CIP), Department of Psychology, Autonomous University of Lisbon, Lisbon, Portugal

**Keywords:** Assessment, Communication, Deaf, Deafness, Management, Pain

## Abstract

**Background:**

Pain is a common reason for seeking out healthcare professionals and support services. However, certain populations, such as people with deafness, may encounter difficulties in effectively communicating their pain; on the other side, health care professionals may also encounter challenges to assess pain in this specific population.

**Aims:**

To describe (a) the state of the research in the field of pain assessment in individuals with deafness; (b) instruments validated; and (b) strategies facilitating the pain communication or assessment in this population.

**Methods:**

A systematic review in accordance with the Preferred Reporting Items for Systematic Reviews and Meta-Analysis guidelines were performed, searching Medline, CINAHL, Scopus, Embase and PsycInfo databases, from their initiation to July 2023. Primary and secondary studies, involving adults with deafness and investigating pain assessment and communication difficulties, facilitators, or barriers, were eligible. The included studies were assessed in their methodological quality with the Quality Assessment for Diverse Studies tool; data extraction and the narrative synthesis was provided by two researchers.

**Results:**

Five studies were included. Two were validation studies, while the remaining were a case report, a case study and a qualitative study. The interRAI Community Health Assessment and the Deafblind Supplement scale have been validated among people with deafness by reporting few psychometric properties; in contrast, instruments well established in the general population (e.g. Visual Analogue Scale) have been assessed in their usability and understandability among individuals with deafness, suggesting their limitations. Some strategies have been documented as facilitating pain communication and assessment: (a) ensuring inclusiveness (the presence of family members as mediators); (b) ensuring the preparedness of healthcare professionals (e.g. in sign language); and (c) making the environment friendly to this population (e.g. removing masks).

**Conclusions:**

The research regarding pain in this population is in its infancy, resulting in limited evidence. In recommending more research capable of establishing the best pain assessment instrument, some strategies emerged for assessing pain in which the minimum standards of care required to offer to this vulnerable population should be considered.

**Supplementary Information:**

The online version contains supplementary material available at 10.1186/s12889-023-16535-5.

## Background

Pain is one of the most common reasons for seeking out healthcare attention, thereby posing a major public health challenge [1]. Although it is a common and universal experience, there are some groups (e.g. children and older people) that are known to experience significant pain disparities, resulting in inequalities in accessing and obtaining appropriate pain care. Consequently, a decreased quality of care and satisfaction, as well as a decreased quality of life have been documented [2–5]. However, despite the increased awareness of social pain disparities, the initiatives for addressing such inequalities have made only modest progress [6, 7]. Specifically, while the still higher prevalence of pain ranging from 9.9% to 50.3% [8] is contrasted by several clinical guidelines targeting different settings [9], ages [10, 11] and clinical conditions [12] no specific pain assessment guidelines have been developed in favour of individuals with deafness despite the recent call for action formulated by the World Health Organization aimed at promoting integrated people-centred ear and hearing care [13]. The invisibility of pain among individuals with deafness contributes to significant healthcare disparities in the Deaf community [14], resulting in fear, mistrust, and frustration in the healthcare encounter [15].

Deafness is defined as a profound or complete loss of the ability to hear from both ears, implying very little or no hearing [16]. The last Global Burden of Disease [17] study reported that 1.57 billion people (95% confidence interval [CI], 1.51–1.64) had hearing loss in 2019 around the world, at least one in five people. Of these, 403.3 million people had moderate- or high-severity hearing loss. Given that age is one of the most important risk factors for hearing loss, as the world population’s age rises, the number of people with hearing loss will increase [17].

Deafness may be a barrier to communication [15] and pain communication [18, 19]; for their part, healthcare professionals might be prevented from understanding and assessing pain that is strictly related to effective communication. As a result, the timely identification of pain intensity and its characteristics, causes, and the degree of relief after treatments is difficult. In this context, patient–healthcare professional communication can also be compromised by other factors such as attitudes and beliefs [20], resulting in pain underestimation or even missed assessment or treatment.

To the best of our knowledge, although some synthesis on communication and inequalities among people with deafness are documented in the literature [21, 22], no summary highlights the state of research on pain assessment among individuals with deafness. Therefore, this study contributes to raising awareness regarding pain in individuals with deafness by exploring in a systematic manner pain assessment and communication evidence with the ultimate intent of identifying recommendations for clinical practice and the research gaps in the field.

### Aims

The study aims were to describe: (a) the state of the research in the field of pain assessment in individuals with deafness; (b) the instruments validated; and (c) the strategies documented as facilitating the pain assessment and/or communication in this population, as documented to date.

## Methods

### Study design, search strategy, and study selection

A systematic review of the literature was performed in accordance with the Preferred Reporting Items for Systematic Reviews and Meta-Analysis (PRISMA) guidelines (Supplementary Table 1) [23]. The Medline, the Cumulative Index to Nursing and Allied Health Literature (CINAHL), Scopus, Embase and PsycInfo databases were searched without time limitations, thus from the database establishment up to 1^st^ of July 2023. The following MeSH terms and/or keywords combined with the Boolean operators AND/OR were used: “deaf”, “deafness”, “pain”, “pain management”, “pain measurement”, “postoperative pain”, and “procedural pain”. The search performed in each database is reported in Table 1.

**Table 1:** Search strategies used in approached databases

Database and search strategy	Results obtained
**PubMed** ("Deafness"[MeSH Terms] OR "Deafness"[All Fields] OR "deaf"[All Fields]) AND ("Pain"[MeSH Terms] OR "Pain Management"[MeSH Terms] OR ("Pain Measurement"[MeSH Terms] OR "pain, procedural"[MeSH Terms] OR "pain, postoperative"[MeSH Terms]) OR "pain communication"[All Fields] OR "pain assessment"[All Fields])	253
**CINAHL** (MH "Deafness + ") OR AB Deafness OR AB deaf) AND ( (MH "Pain + ") OR (MH "Pain Measurement") OR (MH "Postoperative Pain") OR (MH "Pain Management") OR (MH "Pain, Procedural")) OR AB pain communication OR AB pain assessment	41
**Scopus** ( ( TITLE-ABS-KEY ( deaf) AND TITLE-ABS-KEY ( deafness))) AND ( ( TITLE-ABS-KEY ( pain) OR TITLE-ABS-KEY ( pain AND assessment) OR TITLE-ABS-KEY ( pain AND measurement) OR TITLE-ABS-KEY ( pain AND management) OR TITLE-ABS-KEY ( procedural AND pain) OR TITLE-ABS-KEY ( postoperative AND pain) OR TITLE-ABS-KEY ( pain AND communication) OR TITLE-ABS-KEY ( pain AND assessment)))	41
**Embase** ('hearing impairment':ti,ab,kw OR deafness:ti,ab,kw OR deaf:ti,ab,kw) AND (pain:ti,ab,kw OR 'pain assessment':ti,ab,kw OR analgesia:ti,ab,kw OR 'pain measurement':ti,ab,kw OR 'procedural pain':ti,ab,kw OR 'postoperative pain':ti,ab,kw OR 'pain communication':ti,ab,kw)	875
**PsycInfo** (MH "Deafness + ") AND (MH "Pain + ") OR (MH "Pain Measurement") OR (MH "Postoperative Pain") OR (MH "Pain Management") OR (MH "Pain, Procedural")	20
"Deafness" AND (((("Pain"OR "Pain Management") OR "Pain Measurement") OR "Pain, Postoperative") OR "Pain, Procedural")	61

Legend: CINAHL The Cumulative Index to Nursing and Allied Health Literature, Embase The Excerpta Medica

Database; PsycInfo, Psychological Information Database

AB/ABS, abstract; KEY/KW, key words; MH, MeSH term; TI, title

There were eligible: (a) primary and secondary studies; (b) regarding adults (≥ 18 years) with deafness and investigating one or more of the following aspects: (i) pain assessment, evaluation, or measurement; (ii) pain communication difficulties; and (iii) facilitators of, or barriers to, pain assessment and/or management. Therefore, there were excluded: (a) studies involving people with partial hearing loss/hearing impairments or investigating the effectiveness of cochlear implants or other surgical/medical interventions, and other otolaryngologic complications (e.g., tinnitus); (c) published as letters to the editor, or conference abstracts; (d) written in different languages than English and Italian. Grey literature (e.g., unpublished studies) was also excluded.

One researcher (IM) conducted the literature search and evaluated the studies’ eligibility based on title and abstract screening of each publication that emerged. Any doubt in the evaluation regarding eligibility was discussed with a second researcher (AP). The full texts of eligible studies were then retrieved. Two researchers (IM, AP) independently evaluated the full text of each study, and inclusion of the study was decided upon joint agreement, discussing discrepancies with a third researcher (CTP). The reference lists of the included studies were also screened, to identify additional eligible studies.

### Study risk of bias assessment

Considering the heterogeneity of the study designs of the publications retrieved, researchers decided to use the Quality Assessment for Diverse Studies (QuADS) tool, specifically developed to assess the methodological quality of studies when based upon different research approaches [24]. The quality appraisal was performed by one researcher (IM) and checked by another researcher (SC). Findings were used to describe the state of the research in the field.

### Data extraction and analysis

Two researchers (IM, AP) designed a grid for data extraction that was discussed with a third researcher (CTP) and piloted in one study; no changes were required. Thus, the following data were extracted: author(s), year of publication, country, aim(s), study design, main sample characteristics, data collection, and main findings. The data extraction process was conducted independently by two researchers and then agreed upon (IM, AP); in case of studies involving a member of the research team, the data extraction was performed by another member (SC). Discrepancies were discussed with a third researcher (CTP).

The data analysis was performed in two steps: (1) two researchers (IM, SC) summarized the main study features; then, (2) the main findings of the studies included were narratively described in accordance with Popay and colleagues [25] according to the three aims, namely: (a) the state of the research in the field; (b) the instruments validated in this field; and (c) the strategies facilitating pain assessment or communication among individuals with deafness.

## Results

**Fig. 1:**
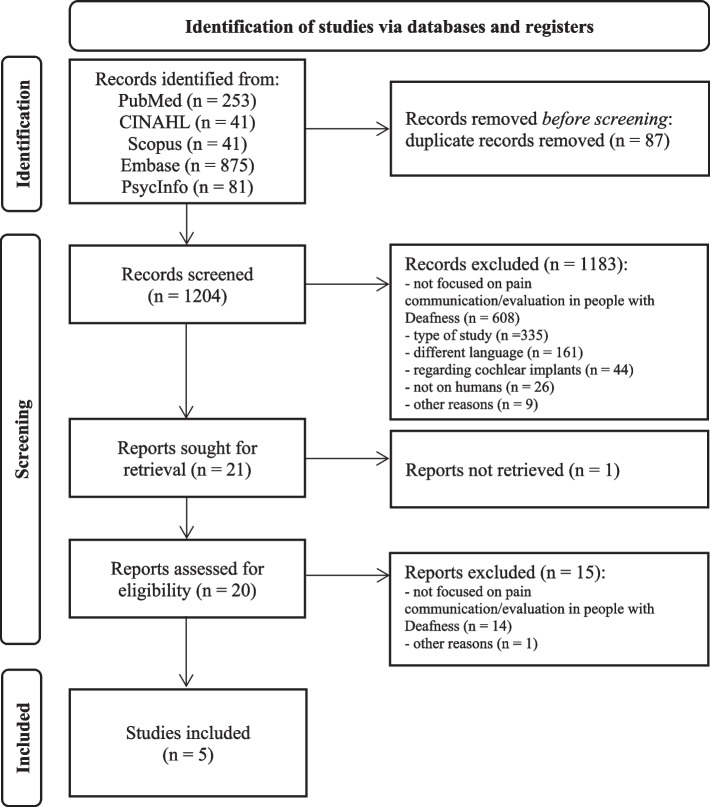
Flowchart for the search and study selection process (following the PRISMA guidelines) [21] Legend: CINAHL, The Cumulative Index to Nursing and Allied Health Literature; Embase, The Excerpta Medica Database; PsycInfo, Psychological Information Database. PRISMA, Preferred Reporting Items for Systematic Reviews and Meta-Analysis guidelines

Starting from 1291 records, there were included five studies as reported in the Fig. 1.

### The state of the research in the field

Among the five studies included (Table 2), two were quantitative validation studies [26, 27], one was a case study implying a community-based participatory research methodology [28], one was a qualitative descriptive study [29], and one was a case report [30]. All studies have been published after 2000, and conducted in North America (three, Canada or the USA) [26–28], in Italy [29] and in India [30].

**Table 2:** Characteristics and main results of the included studies (*n* = 5)

Author(s) (year) Country, Setting	Aim(s), Study design	Participant(s)’ characteristics Data collection	Main results
Allen et al. (2002) [28] USA Setting = Hospice	To understand how the Community-Based Participatory Research is used to develop a means of discussing end-of-life care needs of seniors with deafness Study design = Case study + Community-Based Participatory Research	*n* = 1 person with deafness, with a brain tumour affecting his capacity to communicate in sign language Age = not reported Sex = male The End-of-care Education project of the Minnesota Deaf Community, as a three-year collaboration project, developed the survey covering seven areas: obstacles in obtaining the information; English reading; where people get the information; communication with physicians; decision-making; perspective on death; and understanding about the end-of-life care and hospice Data collection method = the 20 members of the Deaf community purposefully identified participated; the final version of the questionnaire comprised 22 pages, and 64 questions	Clinical implications: - Members of the Deaf community belong to a distinct linguistic and cultural minority group - The most identifiable characteristic is the language: American Sign Language is not a choice – it is the language of the community - Not having the opportunity to access the language is a barrier in the end-of life care decisions - Facial expressions, “non-manuals”, convey meanings: (a) there is one sign for “pain”, but the intensity and its characteristics or variations are communicated by non-manual movements or expressions; (b) rating scales are not always adapted for these aspects, e.g. not all versions of Facial Pain Scale have raised or lowered eyebrows; (c) the numerical rating scales or visual analogue scales are difficult to use by people with deafness because their sign language is not linear and, therefore, they do not identify the left side as a point of start and the right side as an end-point; - The survey needs to be further developed by adding specific questions Research implications: - According to the unique linguistic differences of these populations, researchers and members of the Deaf community should work together
Chowdhry et al. (2016) [30] India Setting = Department of Cardiac Anaesthesiology	To report on difficulties and perioperative management strategies of a patient with deafness and dumbness Study Design = Case report	*n* = 1 patient congenitally deaf who had undergone surgery for double cardiac valve replacement Age = 45 years Sex = male Data collection method = the case is narratively reported	The perioperative management in the patient is challenging - The VAS was used to assess the pain, and the patient was educated by a sign language specialist who was appointed to interact with the patient - Therapeutic strategies were adopted to keep the VAS under 5 Individuals with deafness are at higher risk of receiving inadequate care and information - Pain should be assessed adequately during the perioperative phase - As the patient is unable to convey problems, clinical and objective assessment should be considered - A sign language specialist should be involved to improve pain communication - Once patient is stable, family members should be allowed to spend time with the patient - Non-treated pain could lead to anxiety, insomnia, irritability, aggressive behaviours, stress and sufferance; a rise in heart rates and blood pressure, triggering nausea, vomit and paralytic ileus, ineffective cough and superficial breathing. As a result, undertreated pain may delay mobilization, prolong hospitalization and increase healthcare costs
Dalby et al. (2009) [26] Canada Setting = service provider organizations of government-funded intervener services	To describe the development and pilot testing of the standardized instrument interRAI CHA + DbS to evaluate the needs of deaf-blind people of all social and health service settings Study Design = Validation study	Non-random sample n = 187 participants (response rate 63.8%) 51.7% with acquired blind deafness 48.3% with congenital deaf-blindness Age = mean 42.7 ± 17.8 years (range 18–94) Sex = not reported Data collection method = the new module of DeafBlind Supplement interRAI CHA was developed with extensive feedback from stakeholders and individuals with deaf-blindness Interviews / Feedback on the instrument were gathered from assessors and from 20 in-person interviews with individuals with deaf-blindness or their family members	307 items The Pain Scale measures the frequency and the intensity of the pain to produce a score ranging from 0 (no pain) to 3 (severe daily pain) The Pain Scale obtained a Cronbach α of 0.89 The Pain Scale has been shown to be predictive of pain as measured with the VAS tool
Guthrie et al. (2011) [27] Canada Setting = Canadian Deafblind Association and Canadian National Institute for the Blind	To conduct psychometric testing of the interRAI CHA + DbS instrument, including an assessment of its interrater reliability Study Design = Validation study	Convenience sample n = 44 participants 37.4% had acquired deaf-blindness 63.6% had congenital deaf-blindness Age = mean 40.1 ± 14.8 years (range not reported) Sex = 40.9% male Data collection method = 11 assessors (mean 12 years of experience) from the Canadian Deafblind Association and the Canadian National Institute for the Blind voluntarily participated. All completed the interRAI CHA and DbS as part of a previous project. All assessors attended a 1-day training session Interviews, with or without an intervener or an interpreter. Each participant was interviewed twice within seven days Each assessor participated in an interview to give feedback regarding the assessment itself	150 items, five regarding pain (not reported in the article) 12 subscales Pain Scale obtained a Ƙ value of 0.51 (range 0.18–0.79)
Palese et al. (2011) [29] Italy Setting = not reported	To contribute to the development of knowledge in clinical pain assessment of deaf persons by including the point of view of deaf patients and nurses who cared for them Study Design = Qualitative descriptive	Purposeful sample *n* = 10 nurses with experience in caring of patients with deafness Age = not reported Sex = not reported *n* = 16 participants with deafness Age = mean 43 years Sex = 56% male Data collection method = Focus groups	The scales used by nurses in daily practice were: NRS, VAS, FPS and IPT Participants with deafness evaluated all of them as follows: - NRS: might be ambiguous because in sign language a high mark means “optimal performance” (or no pain) while a low mark means “worse performance” (a lot of pain). Moreover, the tool measures only the “amount” of pain, a characteristic difficult to be understood by individuals with deafness - VAS: might be difficult for individuals with deafness to understand. When the VAS is presented horizontally, it has no meaning for them; the “intensity” or the “amount” is the only pain characteristic under evaluation with this tool: however, people with deafness interpret the questions “how much” and “how many” differently, not understanding what is being asked regarding pain - FPS with six faces: it is not easy to associate the face expression and the pain. Facial expressions, in sign language, are used to communicate emotions without a direct relationship with pain intensity; participants reported that this scale might make them feel ridiculous; - IPT scale: this scale is appreciated because it implies a simple format where the vertical line demonstrates effectively demonstrates the increasing level of the construct evaluated. However, this scale only measures the intensity of pain Regarding methods used to administer the pain evaluation scales, participants reported that: - it is suggested to carefully consider the involvement of relatives as proxies of the patient: however, individuals with deafness want to be understood and treated as independent adults - the use of paper and pen may be appropriate to improve communication; however, older individuals with a low educational level may not be able to write/read, and some might only be able to communicate with sign language. Lipreading and visual contact are considered important, but sometimes healthcare professionals use difficult and sophisticated terms or speak too fast. In some settings (e.g. operating theatre), there are some barriers that impede lipreading (masks) - facial expressions and mimicry are also fundamental in expressing the intensity of the communication, but are sometimes prevented by instrumental devices - the use of a call-bell does not guarantee the possibility to refer pain

Legend: *ADL-H* Activities of Daily Living Hierarchy Scale, *CHESS* Changes in Health, End-Stage Disease, and Signs and Symptoms Scale, *CPS* Cognitive Performance Scale, *DbSI* Deaf-blind Severity Index, *DRS* Depression Rating Scale, *FPS* Faces Pain Scale, *IADL* Instrumental Activities of Daily Living, *IADLI* Instrumental Activities of Daily Living Involvement scale, *IPT* Iowa Pain Thermometer, *ISE* Index of Social Engagement, *NRS* Numerical Rating Scale, *O&M* Orientation and Mobility Capacity Scale, *VAS* Visual Analogue Scale

The two quantitative studies developed and validated a standardized instrument, the interRAI Community Health Assessment (interRAI CHA) and the Deafblind Supplement (DbS), respectively, by assessing some psychometric properties [26, 27]. Allen et al. [28] explored how community-based participatory research develops a means of discussing the end-of-life care needs of seniors with deafness, whereas Palese et al. [29] and Chowdhry et al. [30] aimed at identifying the issues faced by people with deafness when communicating pain.

The studies involved people with deafness [28–30] and/or deaf-blindness [26, 27] and nurses caring for them [29]. Those involved ranged from one adult patient who had undergone cardiothoracic surgery [30] and who was at the end of his life due to a brain tumour [28] up to 187 with acquired or congenital deaf blindness (average age 42.7 years) [26]. Palese and colleagues [29] included 16 patients with deafness (average age 46 years) and ten nurses with experience in caring for them.

The validation studies used in-person interviews with participants [26] by involving an intervenor or an interpreter when required [27]; the case report studies [28, 30] did not report on the data collection used, while Palese et al. [29] conducted video-recorded focus groups where Italian sign language was adopted.

The methodological quality of the included studies as assessed with the QuADS [24] is variable (Supplementary Table 2): in some elements (e.g., research aims, setting and target, study design) most studies provided sufficient or detailed descriptions, while in other (e.g., the justification for the analytic method used), limitations or lacks in the reporting have emerged.

### Instruments for pain assessment

As reported in Table 2, the interRAI CHA and the DbS were first developed by Dalby and colleagues as an adaptation of the interRAI assessment established by the Ontario Ministry of Health and Long-term Care to evaluate the individual needs for both social and health services [26], especially in homecare and primary care settings [27]. The intent of the interRAI CHA and DbS was to better understand the needs of people with deafness and/or blindness, which were not being met equally throughout the population [27]. The instrument included more than 150 items, organized into 10 or more domains, including, for example, activities of daily living, instrumental activities of daily living, cognition, and health conditions [27]. Inside the health condition domain, pain is evaluated with two [26] or five [27] items. Guthrie et al. [27] reported the overall Cronbach’s α ranging from 0.63 to 0.93, while Dalby et al. [26] also explored domain values, in which pain symptoms obtained a Cronbach’s α of 0.89. The mean Ƙ value between the pain and the overall instrument score was 0.51 (0.18–0.79) [27].

The perceptions of individuals with deafness and those of nurses regarding the advantages and disadvantages of pain assessment instruments developed for all people were described by Allen et al. [28] and Palese and colleagues [29]. The Iowa Pain Thermometer (IPT) was the most appreciated and useful tool because the vertical line communicates clearly the increasing symptom intensity, whereas the horizontal orientation of the Visual Analogue Scale (VAS) was not understood by individuals with deafness [29]. In fact, they reported not easily identifying the left side of the VAS line as the starting point, stating that no or little pain is perceived, whereas the opposite is true about the right side [28]. The Facial Pain Scale (FPS) was considered ambiguous because, according to sign language, facial expressions communicate emotions not related to pain [29]. Moreover, this tool was not appreciated because of other factors: for example, not all versions are based on faces with eyebrows, or with raised/lowered eyebrows, which were considered significant in the communication in this context [28]. Additionally, the Numerical Rating Scale (NRS) has also been reported as misunderstandable: in the sign language, a higher number meant a good performance, thus not the “worst pain” as the NRS meant; on the other hand, lower numbers were used to express worse levels of performance, thus contrary to the “lower level of pain” [29].

### Strategies to facilitate pain assessment or communication

Some strategies for improving pain communication and/or facilitating its assessment among people with deafness have emerged across the included studies (Table 2), such as training healthcare professionals in the use of sign language [30], involving sign language specialists [30], and carefully considering the grammatical and sign variances across countries [28]. The presence of family members was also reported as being important for mediating the communication between patient and healthcare professionals [29, 30]. Using facial expressions and mimicry was fundamental, especially in communicating the intensity of emotions or symptoms; visual contact and lipreading were also useful for improving mutual understanding. However, in healthcare settings, some devices (e.g. wearing masks) and the use of complex medical terms have been reported as preventing pain communication and assessment [29]. The use of pencil and paper has been suggested, but older individuals may have received limited education and may be able to communicate only in sign language [29].

## Discussion

### The state of the research in the field

Despite the well-established recommendations regarding how to measure and manage pain across life and in different clinical conditions [31–33] and the initiatives to address pain care disparities and pain in vulnerable social groups [6], individuals with Deafness still represent a neglected population. Primary studies often exclude them, given the complexity of pain measurement (e.g., the need-to-know sign language and to adapt pain assessment measures), whereas, as emerged in our review, those aimed at investigating issues in assessing and communicating pain are rare; moreover, they have involved Deaf cultures according to the range of countries where available studies have been conducted. Therefore, while other factors hindering or promoting inclusiveness, disparities, accessibility and equity in pain measurement and management have been considered by researchers [34], those regarding individuals with deafness require urgent investment.

Studies have been published from 2002 to 2016 and no traces of recent investigations have emerged, suggesting that no priority is given to this field of research. The pandemic crisis has dramatically increased the vulnerability of this population, threatening the communication of their needs (pain included) due to the several barriers imposed by the restrictions employed (e.g. wearing masks, physical distancing) [35]. Moreover, in analysing the included studies at the overall level, some main features have emerged. Firstly, they involved a limited number of patients, ranging from one [28, 30] to 187 [26], which may suggest some difficulties in accessing this population where alliances with associations and representatives are important. The World Health Organization emphasised the need of a person-centred hearing care, that should be considered also in research [13]; therefore, while developing new instruments and/or re-validating those available, assessing the extent of their capacity to be person-centred, thus in line with the recommendations established by the World Health Organization, is strongly suggested.

Secondly, while only two clinical conditions (end of life and cardiac surgery) [28, 30] have been considered, in the remaining studies no specific clinical issues or settings have been targeted, suggesting that currently an inclusive research approach is prevailing by including all patients at risk of pain instead of focusing on certain conditions. Thirdly, studies have involved members of the Deaf community [28], individuals with Deafness, and/or their families [26], as also represented by their associations [27], or nurses [29], indicating that in this field a co-constructive approach is crucial. Fourthly, the data collection is a challenge and an intervenor or interpreters [27] should be involved in ensuring participation and data accuracy. Not lastly, according to the assessment of the studies included, there is a need to improve the methodological quality in this research field to strength the evidence available. All the above-mentioned reflections confirm the underlying complexity of this research field, which should be better supported and promoted across the world in accordance with the diversities in the Deaf culture that might influence pain assessment and communication and thus its effective management. Therefore, this research field is at still need to be expanded [36] both quantitatively and qualitatively.

### Instruments for pain assessment

Despite the challenges in measuring pain in this population, a few specific instruments have been developed and validated to date as compared to the well-documented literature produced in favour of other complex conditions (e.g., dementia care) where several assessment instruments have been produced (up to 28 tools) [12]. Moreover, at the overall level, two different perspectives have been considered in this research field: (a) considering the needs of deaf individuals, thus shaping the tool around their peculiarities by adding specific items assessing pain; and (b) attempting to validate among these individuals those tools used in the general population. Both attempts have considered only unidimensional tools that are lacking in assessing important characteristics of pain according to its multidimensionality [34].

In regard to the first perspective, the interRAI CHA and DbS scale have been validated in order to understand all the needs of people with deafness, not limited to pain [26, 27]. Consequently, it is composed of a high number of items with only a few being intended to assess pain [26, 27], suggesting that this might be used as an initial assessment and should be followed by additional specific instruments or strategies capable of deepening the pain assessment. However, it has been used only in a few countries (e.g., Canada, USA) thus at need to be translated and validated in other countries, with different cultures and languages.

As regards the second perspective, some tools validated among the general population have been considered in terms of their understandability and usability among people with deafness; however, some issues in the sentence structure and in the visual organization have emerged, suggesting the need for prudence in their use with this population [29]. Specifically, the NRS, the VAS, and the FPS have been underlined as presenting visual or structural problems, which may lead to misunderstandings regarding pain. According to the perspective of Italian individuals with deafness, only the IPT tool seems to be valid [29]. Moreover, while reporting their pain, they have been documented to not use terms and/or adjectives commonly used by other patients. Differently, they have been reported to easily communicate the intensity and the site of the pain [37].

In line with the Deaf culture, more research is needed to assess the validity of the IPT across different cultures, while all tools assessing pain should be subjected to more validation studies aimed at establishing their psychometric properties, by also considering different subgroups of individuals, such as children [19], the elderly [38], and foreign people with deafness [28], given the limited investigations performed to date. In future studies, the involvement of people with deafness as individuals and/or as representative associations to better reflect their preferences, values, and need, is strongly recommended.

### Strategies for pain assessment and communication

Strategies aimed at improving pain communication or ensuring an appropriate assessment of pain have emerged across studies as complementary to the use of instruments [29] or alone [30]. At the overall level, these strategies can be summarized as:

- involving and promoting inclusiveness by communicating in sign language: professional trained interpreters [30] or trained volunteers [39] are suggested; moreover, it is also suggested to facilitate the presence of family [29, 30] that may play a mediating role;
- ensuring the preparedness of healthcare professionals by engaging the team in a proper planning and coordination of the care where pain management is a priority [30], or by offering a minimum training in sign language [5, 27];
- making environments friendly toward this population, by removing all communication barriers such as facial masks or shields, as widely used during the Covid-19 pandemic [40]: these aids may interfere with non-verbal communication, such as facial expressions or eye movements, and with lipreading [29, 41], which could be ensured with transparent masks [37].

However, according to the study designs conducted (e.g., case study) [28, 30], these strategies cannot be weighted in terms of the evidence produced, suggesting the need for them to be further scrutinized regarding their effectiveness [36] by involving different study designs and more participants [42].

### Limitations

This review has several limitations. First, the search strategy was applied in different databases to detect published peer-review studies and although the method was double checked, some items can be missed; moreover, grey literature, as well as association websites and government policies, were not eligible. Second, the included studies were based upon different study designs, generating different levels of evidence, thus making difficult the synthesis of useful information and recommendations. Third, there was performed a narrative synthesis of the data extracted from the included studies; with the increased interest in this field of research, a more structured approach in providing a summary of the findings is encouraged.

## Conclusions

Pain communication is a challenge for both individuals with deafness and healthcare professionals; non-verbal individuals are especially at risk of having their pain poorly assessed or managed in their daily care. Therefore, assessing the evidence available by performing a systematic review was considered useful for establishing effective pain recommendations. However, only five studies have been published to date, suggesting that research in this field is in its infancy and suffers a sort of fragmentation where few authors have investigated different aspects, resulting in a limited accumulation of knowledge to address the practice. Establishing this field of research as a priority by providing strong support according to the complex methodologies, allowing the full participation of individuals with deafness and their family/communities and associations/representatives across the world, is recommended.

Instruments validated to date have been developed and shaped according to the needs of individuals with deafness on the one hand, and by assessing the understandability and usability of those tools already used in the general population on the other. In both circumstances, a few validity and reliability properties of unidimensional tools have been assessed, suggesting the need to better consider pain in its multidimensionality by investigating the full properties required for an accurate pain assessment. Consequently, due to the sparse and limited data available, no measurement instrument for the clinical practice can be recommended to date. Therefore, more studies are needed by involving individuals and/or their representatives in the research processes.

Whether complementary to the above-mentioned instruments or alone, some strategies have emerged to facilitate the assessment or the communication of pain among individuals with deafness: involving and promoting inclusiveness (family members, sign language specialists), ensuring the preparedness of healthcare professionals with appropriate training, and making environments friendly toward this population by removing all barriers have been suggested. Although not supported by strong proof of their effectiveness, these simple strategies should be considered the minimum standards of pain care to offer to this vulnerable population.

### Supplementary Information

**Additional file 1:** **Supplementary Table 1.** PRISMA Check list (Page et al., 2021) [23].

**Additional file 2:** **Supplementary Table 2.** Quality assessment of included studies: critical appraisal tools divided by study design [24].

## Data Availability

The datasets used and/or analysed during the current study available from the corresponding author on reasonable request.

## Abbreviations

CINAHL: The Cumulative Index to Nursing and Allied Health Literature
DbS: Deafblind Supplement
FPS: Facial Pain Scale
HCPs: Healthcare Professionals
interRAI CHA: The interRAI Community Health Assessment
IPT: Iowa Pain Thermometer
NRS: Numerical Rating Scale
PRISMA: Preferred Reporting Items for Systematic Reviews and Meta-Analysis
VAS: Visual Analogue Scale
