# Supplementary material for: Facilitating pain assessment and communication in people with deafness: a systematic review
Source: BMC Public Health. 2023 Aug 22;23:1594. doi: 10.1186/s12889-023-16535-5 (PMC10464447; doi:10.1186/s12889-023-16535-5)
Supplement: Supplementary file 2 — Additional file 2: Supplementary Table 2. Quality assessment of included studies: critical appraisal tools divided by study design [24]. [file 12889_2023_16535_MOESM2_ESM.docx]

**Supplementary Table 2.** Quality assessment of included studies: critical appraisal tools divided by study design [25].

| **QuADS criteria** | **Allen et al. (2002) [28]** | **Chowdhry et al. (2016) [30]** | **Dalby et al. (2009) [26]** | **Guthrie et al. (2011) [27]** | **Palese et al. (2011) [29]** |
| --- | --- | --- | --- | --- | --- |
| 1. Theoretical or conceptual underpinning to the research | 1 | 0 | 2 | 2 | 2 |
| 2. Statement of research aim/s | 3 | 1 | 3 | 3 | 3 |
| 3. Clear description of research setting and target population | 1 | 3 | 3 | 3 | 2 |
| 4. The study design is appropriate to address the stated research aim/s | 2 | 3 | 3 | 3 | 3 |
| 5. Appropriate sampling to address the research aim/s | 1 | 0 | 2 | 1 | 2 |
| 6. Rationale for choice of data collection tool/s | 1 | 0 | 3 | 2 | 3 |
| 7. The format and content of data collection tool is appropriate to address the stated research aim/s | 1 | 0 | 3 | 3 | 3 |
| 8. Description of data collection procedure | 1 | 0 | 3 | 3 | 2 |
| 9. Recruitment data provided | 1 | 0 | 2 | 2 | 1 |
| 10. Justification for analytic method selected | 0 | 0 | 1 | 1 | 2 |
| 11. The method of analysis was appropriate to answer the research aim/s | 0 | 0 | 3 | 2 | 3 |
| 12. Evidence that the research stakeholders have been considered in research design or conduct. | 3 | 1 | 2 | 2 | 3 |
| 13. Strengths and limitations critically discussed | 0 | 0 | 1 | 3 | 3 |

Legend: 0, not reported/mentioned; 1, limited reported/explained; 2, basic explanation/description; 3, detailed explanation/description (for detailed information about evaluations, see Harrison et al. (2021) [25].

QUADS, Quality assessment with diverse studies
